# Contextual emotion detection in images using deep learning

**DOI:** 10.3389/frai.2024.1386753

**Published:** 2024-06-17

**Authors:** Fatiha Limami, Boutaina Hdioud, Rachid Oulad Haj Thami

**Affiliations:** Advanced Digital Enterprise Modeling and Information Retrieval (ADMIR) Laboratory, Rabat IT Center, Information Retrieval and Data Analytics Team (IRDA), ENSIAS, Mohammed V University in Rabat, Rabat, Morocco

**Keywords:** computer vision, contextual recognition, EMOTIC, emotion recognition, body language, human–robot communication

## Abstract

**Introduction:**

Computerized sentiment detection, based on artificial intelligence and computer vision, has become essential in recent years. Thanks to developments in deep neural networks, this technology can now account for environmental, social, and cultural factors, as well as facial expressions. We aim to create more empathetic systems for various purposes, from medicine to interpreting emotional interactions on social media.

**Methods:**

To develop this technology, we combined authentic images from various databases, including EMOTIC (ADE20K, MSCOCO), EMODB_SMALL, and FRAMESDB, to train our models. We developed two sophisticated algorithms based on deep learning techniques, DCNN and VGG19. By optimizing the hyperparameters of our models, we analyze context and body language to improve our understanding of human emotions in images. We merge the 26 discrete emotional categories with the three continuous emotional dimensions to identify emotions in context. The proposed pipeline is completed by fusing our models.

**Results:**

We adjusted the parameters to outperform previous methods in capturing various emotions in different contexts. Our study showed that the Sentiment_recognition_model and VGG19_contexte increased mAP by 42.81% and 44.12%, respectively, surpassing the results of previous studies.

**Discussion:**

This groundbreaking research could significantly improve contextual emotion recognition in images. The implications of these promising results are far-reaching, extending to diverse fields such as social robotics, affective computing, human-machine interaction, and human-robot communication.

## 1 Introduction

Emotion recognition has gained a lot of attention recently (Liao et al., 2022) due to its various applications in robotics, education, and human–computer interaction (Alanazi et al., 2023). Previous studies (Zhao et al., 2021; Mamieva et al., 2023) have focused on different human modalities, such as voice, writing, facial expressions, and physiological indicators to recognize emotions. Although facial expressions are useful, our understanding of human emotions (Devaram et al., 2022) can also be influenced by other factors. Therefore, considering the context is essential to correctly decipher the meaning of a particular expression. A smile, for example, may indicate happiness, but depending on the context, it may mask other emotions, such as sarcasm or humiliation. Similarly, a neutral face in a setting can convey a particular emotional meaning.

Some research focused on image recognition, using the methodology recommended by Li et al. (2018). Others, such as Li and Deng (2022), investigated predicting emotional states using visual cues. Cai and Vasconcelos (2017) have also employed a global–local attention technique to extract characteristics from contextual and face areas. Further investigation is necessary to understand the context’s role in emotion detection.

Current emotion identification techniques classify emotions into six basic categories, but these categories need to be improved to account for more complex classifications humans can make. This study, with its potential to revolutionize the field, aims to enhance context-based emotion recognition, a crucial aspect of understanding human behavior and improving human-computer interaction systems. Although progress has been made in this area, there is a clear need for more robust and context-aware emotion detection systems to interpret emotions in various real-world scenarios accurately.

The study will address several key questions, including how to optimize DCNN and VGG19 to enhance the accuracy and performance of sentiment detection systems, how merging discrete emotional categories with continuous emotional dimensions affects the accuracy and context sensitivity of sentiment detection systems, whether integrating diverse emotional databases can enhance the model’s recognition of sentiments across cultural and social contexts, and how the innovative Sentiment_recognition_model and VGG19_context compare to previous methodologies in diverse applications.

Our study delves into the effectiveness of deep convolutional neural networks (DCNN) and VGG19 in image contextual emotion detection. By exploring how our method compares to other advanced methods in different contexts and identifying key contextual factors affecting emotion recognition accuracy, we aim to improve the accuracy of DCNN and VGG19 significantly. Our results provide valuable insights into developing more adaptive emotion detection systems, potentially revolutionizing the accuracy of emotion recognition in various environmental conditions.

To achieve this, we build two advanced deep learning-based models: the Sentiment_recognition_model deep convolutional neural networks (DCNN) and the VGG19_context model. These models are pre-trained on real-world images from the EMOTIC dataset, feature attention mechanisms, and contextual feature extraction methods that enable a more precise and nuanced understanding of emotions. By integrating continuous dimensions and discrete categories in emotion description and considering both body language and environmental context, we aim to create a more comprehensive and accurate system for emotion recognition in diverse contexts. Our approach for recognizing emotions combines commonly used continuous dimensions such as valence, arousal, and dominance with 26 discrete emotion categories. We tested our method on the EMOTIC dataset, designed explicitly for contextual emotion recognition. Our method considers context’s crucial role in detecting emotional states. The research aims to improve the identification of emotions in images by highlighting the importance of contextual information. This application has implications in affective computing, social robotics, and human-computer interaction. We outperformed earlier research, with the DCNN Sentiment_recognition_model reaching an average accuracy (mAP) of 78.39% and the VGG19_contexte system reaching 79.60%. These represent considerable gains in accuracy.

The article is organized into several sections: a review of previous studies on emotion recognition, an in-depth explanation of our systems and how they are implemented, an experimental phase with model architecture and results, and a conclusion that summarizes our findings and suggests future lines of inquiry.

## 2 Related work

Recent research in emotion recognition and computer vision has seen significant strides. Liao et al. (2022) introduced FERGCN, significantly advancing facial expression detection, particularly in challenging conditions. It excels in managing complexities like occlusions and pose changes. Ren et al. (2016) contributed to object detection with their efficient Region Proposal Network, achieving high accuracy and real-time processing on GPUs. Devaram et al. (2022) proposed LEMON, a compact, effective system for emotion recognition in assistive robotics, suitable for limited hardware resources. Li et al. (2018) developed EAC-Net to enhance Action Unit detection in facial images, eliminating the need for preprocessing. A comprehensive survey by Li and Deng (2022) addressed critical challenges in deep facial expression recognition. Cai and Vasconcelos (2017) improved object detection accuracy with Cascade R-CNN, training detectors at increasing IoU thresholds.

EfficientDet by Tan et al. (2020) improved object detection efficiency with compound scaling and BiFPN. DeepFaceEditing by Chen et al. (2021) enabled precise control in facial image synthesis using disentanglement techniques. He et al. (2014) addressed CNN limitations with SPP-net, enhancing classification accuracy through spatial pyramid pooling. Ma et al. (2023) Presented VTFF, utilizing global self-attention and attentional selective fusion for optimal facial expression recognition. Huang et al. (2023) used deep neural networks for facial emotion recognition, focusing on critical features and improving accuracy through transfer learning.

In understanding emotions, the Valence-Arousal-Dominance model has been prominent, with studies by Yang et al. (2016), (Lin et al., 2017), and Park et al. (2021) utilizing it. This model characterizes emotions in dominance, arousal, and valence. Du et al. (2014) introduced a more complex 21-category framework for facial emotions, capturing combinations like “happily surprised.”

Emotion recognition has also extended beyond facial features. Elfwing et al. (2018) considered shoulder position as an indicator for recognizing basic emotions, while Santhoshkumar and Kalaiselvi Geetha (2019) developed a system for identifying 15 emotions and evaluated them on an emotion dataset from the University of YORK, and Ahmed et al. (2020) used body postures for emotion recognition. Jianhua et al. (2020) explored emotion recognition using multichannel EEG and multimodal physiological signals using different feature extraction and reduction methods and machine learning classifier designs.

Contextual information has become crucial in emotion recognition, as shown by Lee et al. (n.d.) and Kosti et al. (2019). Their models use two-stream fusion networks focusing on facial/body modality or context. Le et al. (2021) employed a global–local attention mechanism, and Feldman and Greenway (2021) explored emotional responses to work interruptions, finding they could evoke positive or neutral emotions alongside negative ones. They identify subjective temporal perceptions and contextual factors as critical influences, enriching existing theories on organizational interruptions. Zhang et al. (2020) explores EEG-based emotion recognition methods, covering feature extraction techniques, machine learning classifier designs and the correlation between EEG rhythms and emotions. It also compares various ML and deep learning algorithms. Hoang et al. (2021) have identified three contextual elements: the individual, situation, and culture. These elements play a significant role in determining a person’s emotions. In light of this, we have developed two deep learning systems to improve emotion recognition accuracy by incorporating contextual information.

Previous studies on emotion recognition have explored different architectures to achieve optimal performance in this domain. However, these studies have certain limitations that require further investigation. For example, some studies have focused solely on basic emotion recognition tasks without considering the contextual factors influencing emotional states in real-world scenarios. Furthermore, most of these approaches depend on standard reference datasets, which may only partially reflect the diversity of emotions and contextual nuances encountered in everyday life. These limitations underscore the need for more context-sensitive emotion detection systems that can effectively adapt to diverse real-world environments. In this study, we aim to fill this gap by examining the performance of deep convolutional neural networks in detecting context-sensitive emotions from real-world images and diverse datasets. Unlike previous approaches, which have mainly focused on essential emotion recognition, our study seeks to explore how contextual information can improve the accuracy and robustness of emotion detection models. Our research aims to provide a nuanced understanding of how emotion recognition is influenced by context.

## 3 Proposed method

In recent studies, researchers have made great strides toward enhancing model performance by incorporating contextual data, like human locations and movements (Mittal et al., 2020; Nguyen et al., 2021). Notwithstanding these advances, our approach is unique in that it simultaneously learns to identify contextual and physical emotions in photos of people exhibiting a range of emotional states while performing different jobs. The performance of neural networks in processing information is greatly enhanced when considering the interdependence of environmental and physiological contexts. When describing emotions with discrete variables and continuous dimensions, this performance is very pertinent. Furthermore, we employ three continuous dimensions and 26 discrete variable keywords to precisely detect sentimental and emotional states. Based on their unique features and advantages, we compared these models with other architectures, such as ResNet and Transformers.

A DCNN, with its multiple convolutional, clustering, and fully connected layers, is an ideal choice for image classification tasks. It excels in hierarchical feature learning from raw pixel values, making it a practical and effective tool for image recognition and classification.

VGG19 is a modified version of the VGG model characterized by its deep, 19-layer architecture composed mainly of 3 × 3 convolutional filters. Its uniform architecture makes it easy to understand and implement. Thanks to its deep, narrow structure, it works well for various visual recognition tasks.

ResNet, introducing ignored connections or residual blocks, allows the network to learn residual mappings instead of directly approximating the desired underlying mapping. While ResNet is a powerful tool, it can be more complex to implement and train than shallower architectures such as VGG19. Additionally, deep ResNet architectures can face computational constraints during training, an essential consideration for researchers and data scientists.

Transformers use a self-attention mechanism to evaluate the meaning of different input tokens. They consist of encoder and decoder layers and were initially developed for natural language processing (NLP) tasks. Although transformers have enjoyed success in NLP, their application to image-based tasks such as emotion recognition has yet to be explored. They may require significant computational resources for training, and their performance could be sensitive to hyperparameter parameters.

**Figure 1:**
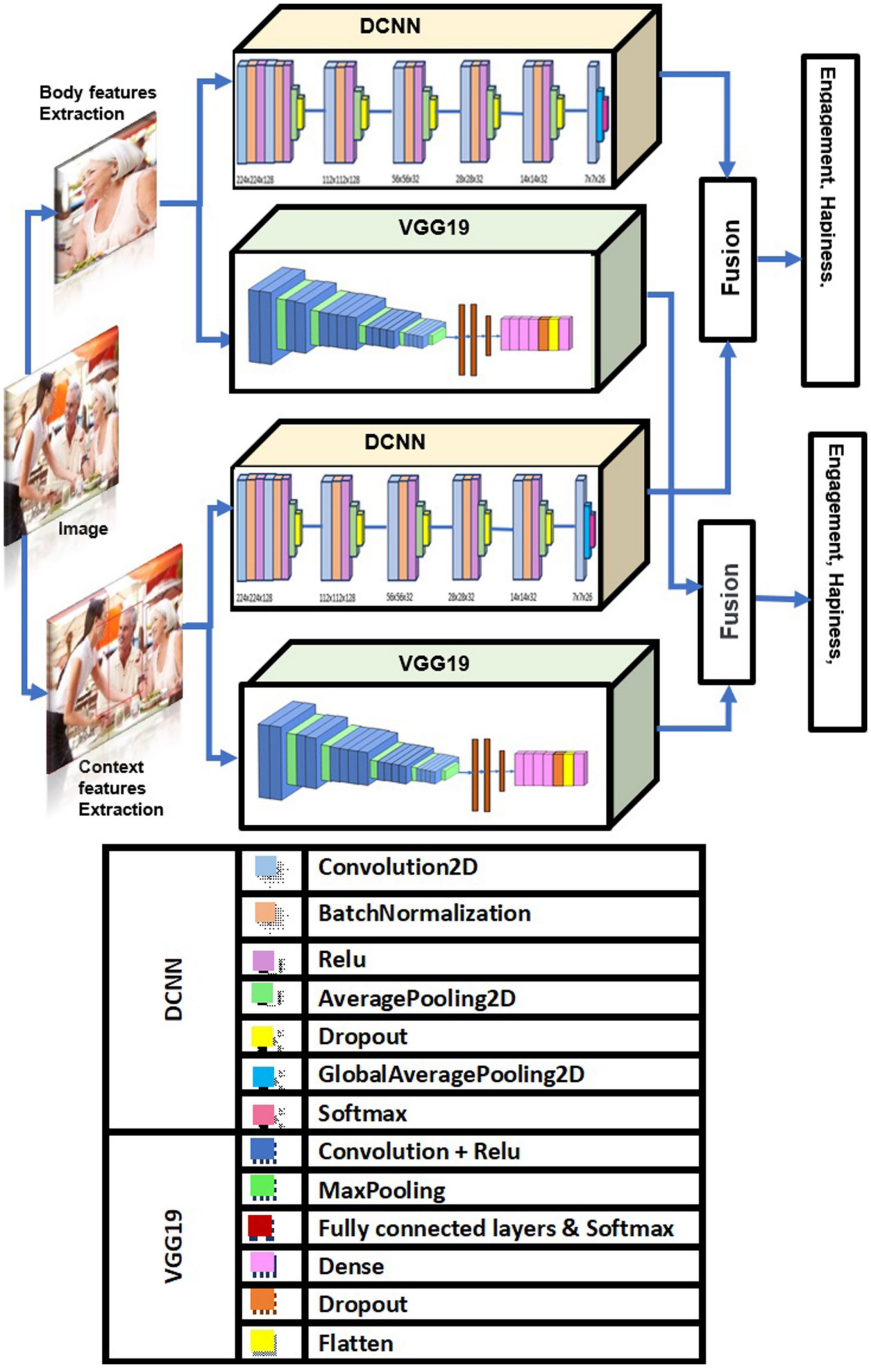
Proposed method for contextual emotion detection. Images reproduced from Papers With Code- Frames Dataset (https://paperswithcode.com/dataset/frames-dataset), licensed under CC BY-SA.

We used DCNN and VGG19 in contextual emotion recognition, as they can efficiently extract image features. Figure 1 above illustrates the pipeline of our proposed solution and shows how our method seamlessly integrates contextual and physiological data to improve model performance.

We aim to improve emotion recognition by using body modalities and sociodynamic interagent interactions to infer contextual emotion more accurately than earlier techniques. We employ a standardized pipeline for all machine learning and deep learning tasks to achieve this. This pipeline includes data collection, preprocessing, feature extraction, model tuning, and sentiment categorization. Figure 1 illustrates the entire process. For our experiment, we use the EMOTIC dataset (Li et al., 2018). During preprocessing, we resize each image to meet the input shape requirements for the proposed DCNN-based model, VGG19. After scaling down the images to 0–255, they are reduced to either 224 x 224 x 3 or 150 x 150 x 3 pixels, depending on the model used. Each image in the dataset has the same size and a pixel value between 0 and 255. Our method involves calculating the probability of each category of variables, including discrete and continuous variables, to predict the emotions of the principal agent. This is done after obtaining their body characteristics (B) and contextual cues (C). We calculate this probability using a formula:

- B: Body Features Extracted using convolutional layers tailored to recognize specific body postures from image data.
- C: Context Features Extracted by different convolutional layers that capture background elements or situational details from the same inputs.
- P(Y): This represents the probability of predicting the principal agent’s emotion (Y). We are interested in determining the overall probability.
- *P*(*Y*/*B*): This is the conditional probability of observing emotion Y, given the principal agent’s body characteristics (B). It represents the likelihood of predicting a specific emotion based solely on the individual’s body characteristics.
- *P*(*Y*/*C*): This is the conditional probability of observing emotion Y given the contextual cues (C) present. It represents how likely it is to predict a specific emotion based solely on contextual information.
- *P*(*B*∩*C*): This term represents the joint probability of both body characteristics (B) and contextual cues (C) occurring together. It reflects the probability of observing both types of information simultaneously.

The formula P(Y) = P(Y/B) + P(Y/C) − P(B∩C) is a sophisticated calculation that intricately measures the overall probability of predicting the emotion Y. It does so by summing the probabilities of predicting Y based on body characteristics and contextual cues separately and subtracting the probability of both occurring together to avoid double-counting.

The function F in our neural network produces the output of the fully connected layer. This function maps an input X, consisting of N inputs (x_1_, …, x_N_) $\in {\mathbb{R}}^{N}$ , to a prediction Y, consisting of M outputs (y_1_, …, y_M_) $\in {\mathbb{R}}^{M}$ , where M = 29.

The function is defined as:


$$F:{\mathbb{R}}^{N}\to {\mathbb{R}}^{M},$$

Y = F(X) = Relu ( $\;\overset{N}{\underset{i=1}{\sum }}\omega_{i}x_{i}+b_{i}$ ), where $\omega_{i}$ represents the weight of input xi and $b_{i}$ represents its bias. The number of inputs N is determined by the size of the input image, which has a width W, a height H, and three color channels.


$$N=\left(\mathrm{W,H,}3\right)=\{\begin{array}{c} \left(224,224,3\right)\;forDCNN\\ \left(150,150,3\right)\;for\;VGG. \end{array}$$

The activation function Relu is defined as follows:


$$\;\mathrm{Relu}\left(x\right)=\{\begin{array}{c} 0\;if\;x<0\\ 1\;if\;x\geq 0 \end{array}$$

We estimate:

${\hat{y}}_{i}^{d}$ : the production for discrete variables “I”

$\;\;y_{i}^{d}$ : the real production for discrete variables “I”

${\hat{y}}_{i}^{c}$ : the estimated production for continuous values “I”

$y_{i}^{c}$ : the real production for continuous values “I”

$L_{d}$ : the loss function for discrete variables

$L_{c}$ : the loss function for continuous values

During the training of our neural network, we use the categorical_crossentropy loss function, i.e.:


$$L_{d}\left(y^{d},{\hat{y}}^{d}\right)=-\frac{1}{N}\overset{N-1}{\underset{i=0}{\sum }}y_{i}^{d}*\log{\hat{y}}_{i}^{d}\;$$

$$L_{c}\;\left(y^{c},{\hat{y}}^{c}\right)=-\frac{1}{N}\overset{N-1}{\underset{i=0}{\sum }}y_{i}^{c}*\log{\hat{y}}_{i}^{c}$$

A combination of the $L_{d}$ and $L_{c}$ loss functions is minimized by the network architecture. The combined loss function of it is as follows:


$$L_{comb}\;\left({\hat{y}}_{i}^{c},{\hat{y}}_{i}^{d}\right)=\min\left(L_{c}\right)+\min\left(L_{d}\right)$$

Optimization is performed using the Adam optimization method and the categorical cross-entropy loss function. A final prediction is obtained by using the activation function Ψ, which combines two neural networks:


$$\Psi \left(x\right)=\left\{\begin{array}{c} \;{\hat{y}}^{c}\;\forall x\in {\mathbb{R}}^{N}\\ {\hat{y}}^{d}\;\forall x\in {\mathbb{R}}^{N}\; \end{array}\right.\;$$

Our research focuses on improving the precision of emotion identification. Differences in lighting, positions, perspectives, and levels of contextual information present the main challenge for human emotion recognition. The context-aware attention network aims to solve this problem by merging body features and contextual emotional features. All images’ fusion features and emotional scores are pooled together to predict human emotions in an image.

We have introduced a new dataset called EMOTIC, which combines the EMOTIC (ADE20K, MSCOCO), EMODB_SMALL, and FRAMESDB datasets, containing images of individuals in various natural environments, together with annotations on their perceived emotions. This dataset is intended to enhance research on context-based emotion recognition. The EMOTIC dataset includes 26 discrete emotion categories and continuous dimensions of valence, arousal, and dominance, rated on a scale of 1 to 10, representing bodily and contextual emotions. We carried out a comprehensive analysis of the dataset using statistical and algorithmic methods and an analysis of the annotations.

Therefore, we are working on both dimensions using two different architectures. One is based on the DCNN architecture and the other on the VGG19 architecture. We trained our models for emotion recognition to automatically recognize emotional states on this new dataset. This model combines information from the bounding box surrounding an individual with contextual information extracted from the scene. We will compare the results of our architectures with those of previous research conducted on these emotional categories. Our research demonstrates that scene context provides critical information for the automatic recognition of emotional states and encourages future research.

## 4 Experiments

### 4.1 Dataset statistics

The EMOTIC (Kosti et al., 2017) database is a vast collection of images that have been sourced from the MSCOCO (Lin et al., 2015) and ADE20K (Zhou et al., 2018) datasets. This database has been created to assist researchers and developers in creating effective emotion identification systems. To provide a comprehensive and diverse image collection for emotion identification research, we have combined images from the EMOTIC, Emodb_small, and Framesdb datasets.

1. The ADE20K (Zhou et al., 2018) is a scene analysis dataset that segments objects and regions in images and offers over 20,000 images with pixel-level annotations for 150 objects and 50 scene categories. It is widely used for semantic segmentation and scene understanding tasks.
2. Microsoft Common Objects in Context (MSCOCO) (Lin et al., 2015) is a renowned reference dataset for image captioning, object detection, and segmentation tasks, comprising over 200,000 images from 80 object categories. It includes detailed annotations such as object delimitation boxes and captions.
3. Emodb_small is a subset of the Extended Cohn-Kanade (CK+) (Lucey et al., 2010) database, featuring grayscale facial images of 15 individuals displaying six basic emotions (anger, disgust, fear, happiness, sadness and surprise). Each image comes with manually labeled emotion annotations.
4. Framesdb is a dataset for emotional video analysis, offering videos from various sources and annotations for affective dimensions like valence, arousal, and dominance. It is suitable for tasks such as emotion recognition and video summarization.

**Figure 2:**
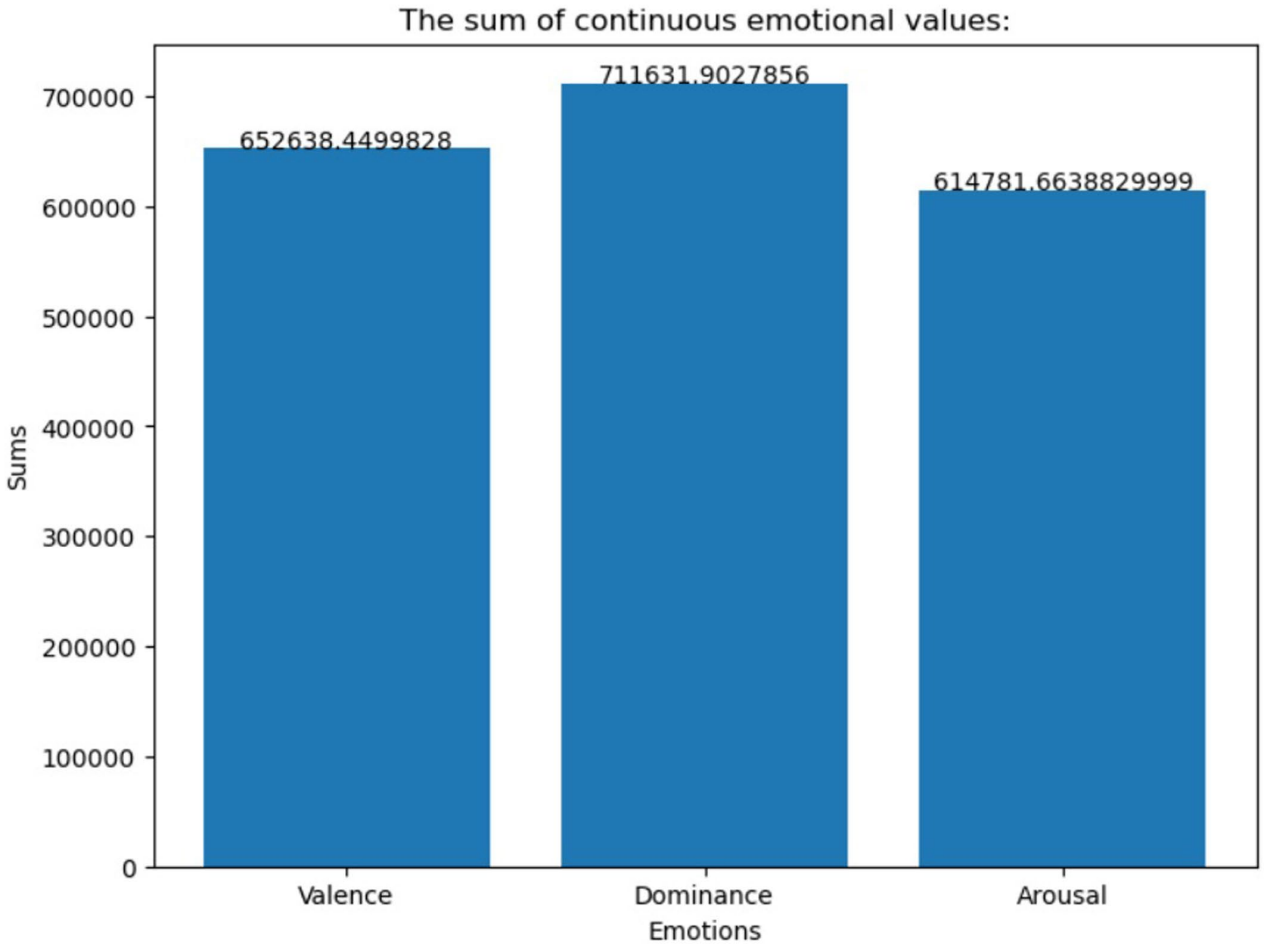
The number of images for the continuous dimensions of valence, arousal, and dominance in the EMOTIC dataset.

**Figure 3:**
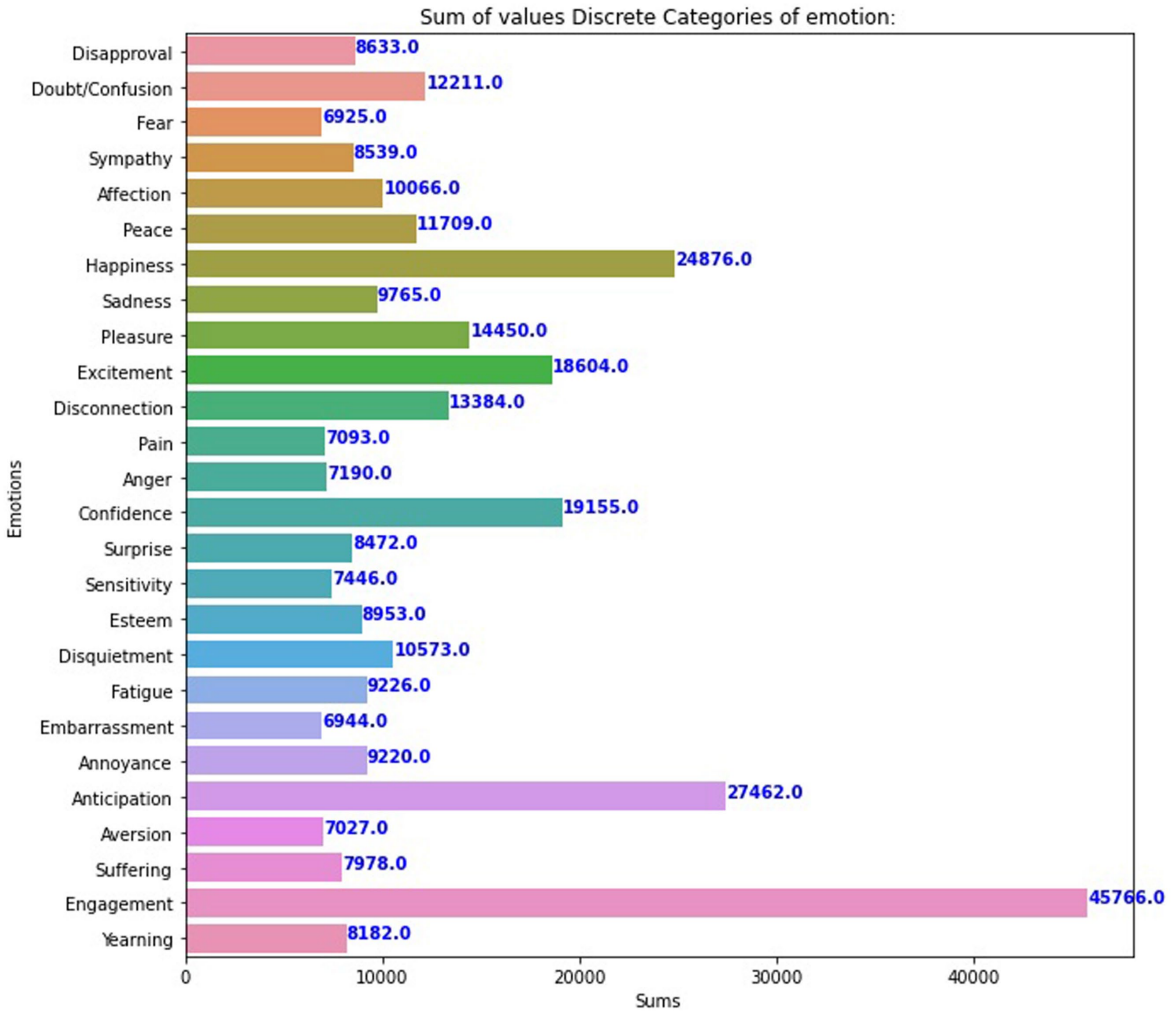
The number of images for each discrete Emotic variable category.

The EMOTIC database is a collection of around 116,034 images that depict a wide range of events and activities. The statistics are then shown in a graph, as seen in Figures 2, 3; it includes labels for 26 emotions, making it a valuable resource for developing and testing emotion detection models in various visual contexts. Before use, the dataset undergoes several preprocessing operations, such as normalization, scaling, and rescaling, to optimize the inputs for different models. The dataset is typically split into training, validation, and testing sets, following a standard ratio of 80% for training and 20% for validation and testing. By combining diverse datasets into EMOTIC, we aim to create more nuanced and practical models for understanding human emotions through visual cues.

For this project, we used two models: Sentiment_recognition_model architecture and VGG19_contexte. We will present the results of each model and evaluate them to determine which is more reliable.

### 4.2 Architecture of the DCNN model

**Figure 4:**
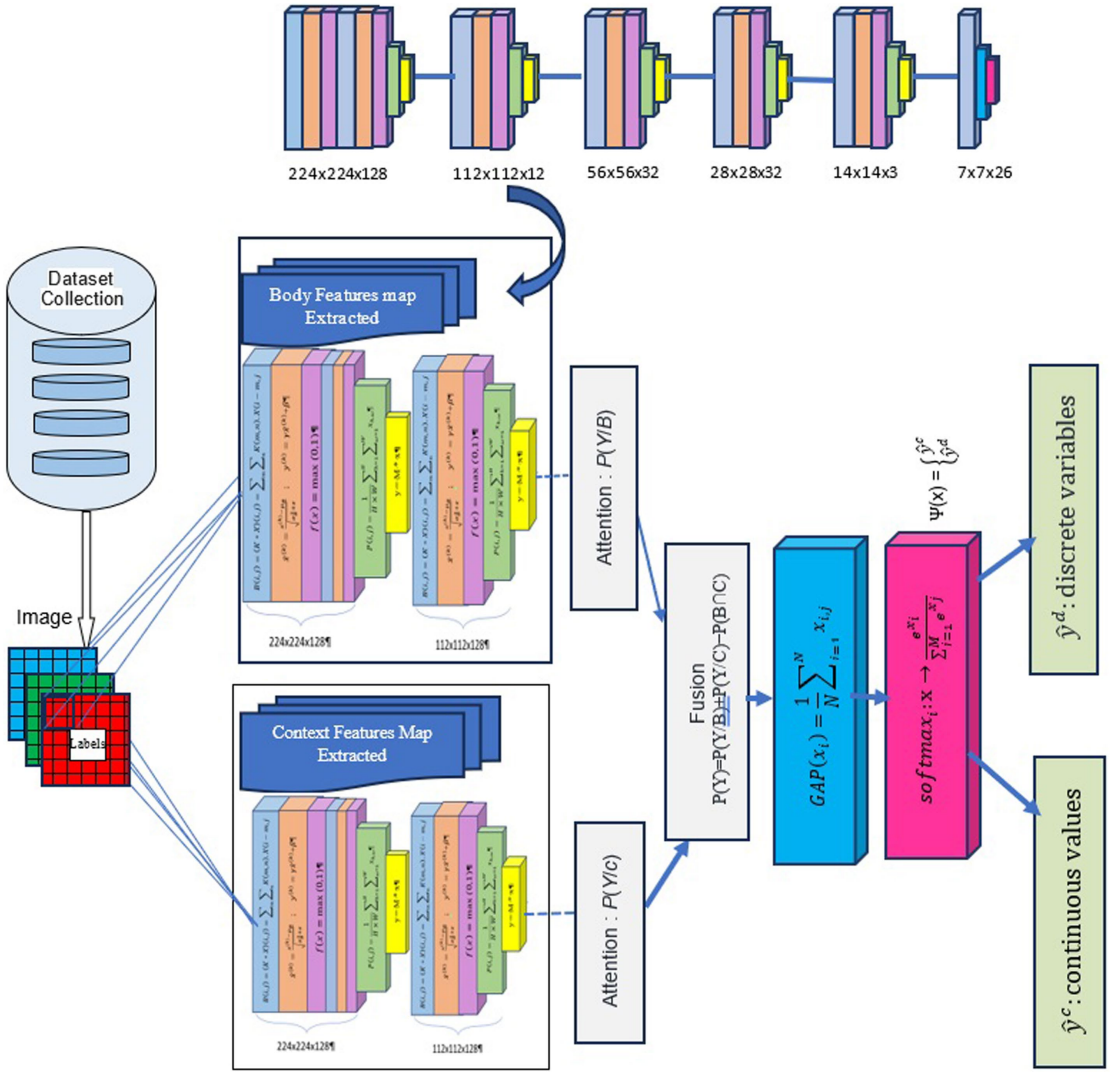
Architecture of the DCNN Model: Sentiment_recognition_model.

The Sentiment_recognition_model The is a deep convolutional neural network (DCNN) created for emotion recognition with the Keras library. This model can process images of size (224, 224, 3) and identifies 26 different emotion classes. The architecture prente in Figure 4 starts with a convolutional layer that has adjustable filters through hyperparameter tuning. In the convolutional neural network, the first stage involves a convolutional layer that processes the input image or feature maps. This layer applies multiple filters, each performing a convolution operation defined by the formula


$$B\left(i,j\right)=\left(K*X\right)\left(i,j\right)=\underset{m}{\sum }\underset{n}{\sum }K\left(m,n\right).X\left(i-m,j-n\right)$$

Where B(i,j) represents the body feature map extracted at position (i,j). B represents the global context feature map. K denotes the filter kernel, X represents the input image or feature map, and the * symbol means the convolution operation. This operation slides each filter over the input, multiplying and summing to produce body feature maps emphasizing specific image features. Following convolution, batch normalization is used to normalize the output of a previous activation layer by subtracting the batch mean and dividing it by the batch standard deviation. This efficient process aims to improve training speed and stability by reducing internal covariate shifts. The operation is defined as:

Where B(i,j) represents the body feature map extracted at position (i,j). B represents the global context feature map. K denotes the filter kernel, X represents the input image or feature map, and the * symbol represents the convolution operation. This operation slides each filter over the input, multiplying and summing to produce body feature maps emphasizing specific image features. Following convolution, batch normalization is used to normalize the output of a previous activation layer by subtracting the batch mean and dividing it by the batch standard deviation. This efficient process aims to improve the training speed and stability by reducing internal covariate shifts. The operation defined as:


$${\hat{x}}^{\left(k\right)}=\frac{x^{\left(k\right)}-\mu_{B}}{\sqrt{\sigma_{B}^{2}+\varepsilon }};y^{\left(k\right)}=\gamma {\hat{x}}^{\left(k\right)}+\beta$$

Where $x^{\left(k\right)}$ is the input to a neuron, $\mu_{B}$ and $\sigma_{B}^{2}$ are the mean and variance calculated over the batch, ε is a small constant to prevent division by zero, and γ and β are parameters to be learned. This step aims to improve the efficiency and reliability of the model. The ReLU activation function is applied next, serving the purpose of introducing non-linear processing to the network. It allows only positive values to pass through, helping the model learn complex patterns. Pooling layers then reduce the spatial dimensions of the feature maps. Average pooling calculates the average of all elements within a specific window size, as per the formula:


$$P\left(i,j\right)=\frac{1}{H\times W}\overset{H}{\underset{h=1}{\sum }}\overset{W}{\underset{\omega =1}{\sum }}x_{h,\omega }$$

where H × W is the size of the pooling window, and $x_{h,\omega }$ are the elements of the feature map covered by the pooling window. This function reduces the output size and helps detect features invariant to scale and orientation. Dropout is incorporated as a regularization method, randomly turning off a subset of neurons during training to prevent overfitting. This layer is mathematically represented as y = M * x, where x is the input vector, y is the output vector, and M entries are 0 with probability p (dropout rate) and 1 with probability 1 − p. Finally, the network concludes with a global average pooling (GAP) layer, simplifying each feature map to a single numerical average with:


$$GAP\left(x_{i}\right)=\frac{1}{N}\overset{N}{\underset{j=1}{\sum }}x_{i,j}$$

where N is the number of elements in each feature map, and $x_{i}$ are the elements of the i-th feature map. GAP flattens the feature maps before they are fed into the final classification layer, where a softmax activation function calculates the probabilities for each class using


$$\sigma \left(Z_{i}\right)=\frac{e^{Z_{i}}}{\sum_{j}e^{Z_{j}}}$$

where ${Ζ}_{i}\;$ are the inputs to the softmax function from the last layer outputs.

The model’s performance is optimized using Keras Tuner, which conducts a random search to determine the best combination of hyperparameters, such as the number of layers, filters, and dropout rate, as well as the learning rate for the Adam optimizer. The model is compiled with categorical cross-entropy loss and evaluated on accuracy and precision metrics. The tuner aims to maximize validation precision through multiple trials and executions, ultimately seeking the most effective model configuration for emotion recognition. The tuner performs a random search for hyperparameters, conducting a maximum of 5 trials and two runs per trial. The results of these settings will be stored. The tuner looks for the best model configuration by using training data, validation data, and epoch count.

The Sentiment_recognition_model for continuous dimensions begins with a Convolution2D layer with 224 filters. It applies a series of learnable filters (kernels) to the previous layer’s input image or context feature maps. Each filter in the Conv2D layer slides over the input data and performs element-wise multiplication followed by a summation, outputting a new context feature map that highlights specific features from the input. Mathematically, this can be represented as:


$$C\left(i,j\right)=\left(K*X\right)\left(i,j\right)=\underset{m}{\sum }\underset{n}{\sum }K\left(m,n\right).X\left(i-m,j-n\right)$$

C(i,j) is the context feature map extracted at position (i,j), knowing that C is the extracted global context feature map, is the filter kernel, is the input image or context feature map, and ∗ denotes the convolution operation. They were followed by batch normalization. This model is repeated several times, and each convolutional block uses ReLU activation. After that, AveragePooling2D and a Dropout layer are added to reduce overfitting, with the dropout rate set at 0.5. The model gradually decreases the number of filters in the following layers (128, 64) and finally ends with a Convolution2D layer corresponding to the three classes: Valence, Arousal, and Dominance. GlobalAveragePooling2D is then added, followed by a softmax activation for classification purposes. The model is compiled using the Adam optimizer and the categorical cross-entropy loss function, focusing on accuracy as the metric.

### 4.3 The architecture of the VGG19 model

Our model, VGG19_contexte, is a deep convolutional neural network (DCNN) customized to identify different emotions. It utilizes Keras and is inspired by the VGG19 architecture. The model aims to categorize 26 distinct emotions, processing images of 150×150 pixels in RGB format.

The model employs a linear stack of layers, starting with a sequential API from Keras. The initial layer is ZeroPadding2D, which pads the input image for consistent size after convolutions.

The ZeroPadding2D layer in a convolutional neural network is a precise tool that adds rows and columns of zeros around the input tensor’s spatial dimensions. This meticulous process is typically done to control the spatial dimensions of the output feature maps after convolutional operations. Let us denote the input tensor as 𝑋 with spatial dimensions, where 𝐻 represents the height, 𝑊 represents the width, and D represents the number of channels.

Mathematically, the ZeroPadding2D operation can be represented as follows:

Given an input tensor 𝑋 of shape H×W×D and a padding configuration of (t1, t2) for the height and (t3,t4) for the width, where t1,t2,t3,t4 represent the number of rows/columns of zeros to add to the top/bottom/left/right of the input tensor, respectively:

The output tensor 𝑌 after applying ZeroPadding2D can be calculated as:


$$Y_{i,j,k}=\left\{\begin{array}{c} 0\;if\;i\langle t1\;or\;i\rangle H+t2\;or\;j<t3\;or\;j\geq W+T4\\ X_{i-t1,j-t3,k}\;otherwise\; \end{array}\right.$$

Where:

-
$Y_{i,j,k}\;$ represents the value at position ( i,j,k ) in the output tensor Y .
-
$X_{i,j,k}$ represents the value at position ( i,j,k ) in the input tensor 𝑋.

The padding configuration specifies zero padding around the input tensor’s spatial dimensions, preserving input tensor data and controlling output dimensions.

The output feature map Y after applying the convolution operation can be calculated as:


$$Y_{i,j,k}=\overset{D}{\underset{d-1}{\sum }}\overset{K}{\underset{m-1}{\sum }}\overset{K}{\underset{n-1}{\sum }}X_{\left(i+m-1\right),\left(j+n-1\right),d}\times F_{i,m,n,d,k}+b_{k}$$

Where:

-
$X_{\left(i+m-1\right),\left(j+n-1\right),d}$ represents the value at position ( i+m−1,j+n−1,d ) in the input feature map X .
-
$F_{i,m,n,d,k}$ represents the value of the filter at position ( m,n,d,k ) in the set of filters $F_{i}$ .
-
$b_{k}$ represents the bias term associated with the k -th output channel.

Convolutional Neural Network (CNN) involves computing each position ( i , j ) in the output feature maps of the set of filters $F_{i}$ . The computation results in a collection of output feature maps with spatial dimensions determined by the filter size and the padding/striding configurations. These output feature maps have D_out_ output channels. The CNN architecture contains several Convolution2D layers, each using ReLU activation. The filters of these layers, ranging from 32 to 256, are determined based on hyperparameters, which serve as the backbone for feature extraction. Additional ZeroPadding2D layers are used in the CNN to maintain the spatial dimensions. Unlike the typical VGG19 model, this architecture does not follow convolutional blocks with pooling layers.

The output feature map Y after applying the max pooling operation can be calculated as:


$$Y_{i,j,k}=\max_{m-1}^{p}\max_{n-1}^{p}X_{\left(i-1\right).P+m,\left(j-1\right).P+n,k}$$

Where:

-
$X_{\left(i-1\right)P+m,\left(j-1\right).P+n,k}$ represents the value at position ( (i−1).P+m,(j−1).P+n,k ) in the input feature maps X .
-
max represents the maximum operation applied over the pooling window size P×P .

This operation is performed for each non-overlapping spatial region defined by the pooling window, resulting in output feature maps with reduced spatial dimensions $\frac{H}{P}\times \frac{W}{P}\times D$ .

The model’s performance is improved by strategically designing the network architecture. After convolutional layers, a Flatten layer reshapes the 2D feature maps into a 1D vector. Two Dense layers with ReLU activation follow this, with units determined via hyperparameter tuning (8, 16, or 32 units). Dropout layers follow each Dense layer to prevent overfitting, with their rates also determined through hyperparameter tuning. The model’s adaptability is further demonstrated by the concluding layer, a Dense layer with 26 units, each representing an emotion class, and using softmax activation to output class probabilities. The model is compiled using the Adam optimizer, with a learning rate optimized through hyperparameter tuning. The loss function is categorical cross-entropy, suitable for this multiclass classification task. This approach to hyperparameter tuning ensures the model’s optimal performance for the specific emotion recognition task.

A RandomSearch tuner from Keras Tuner is employed for hyperparameter optimization, aiming to maximize validation precision. This process involves 2 trials, each with 2 executions, to find the optimal model configuration. This model exemplifies a sophisticated and practical approach to emotion recognition. It leverages the strengths of CNNs and the adaptability of hyperparameter tuning to ensure optimal performance for this specific application.

**Figure 5:**
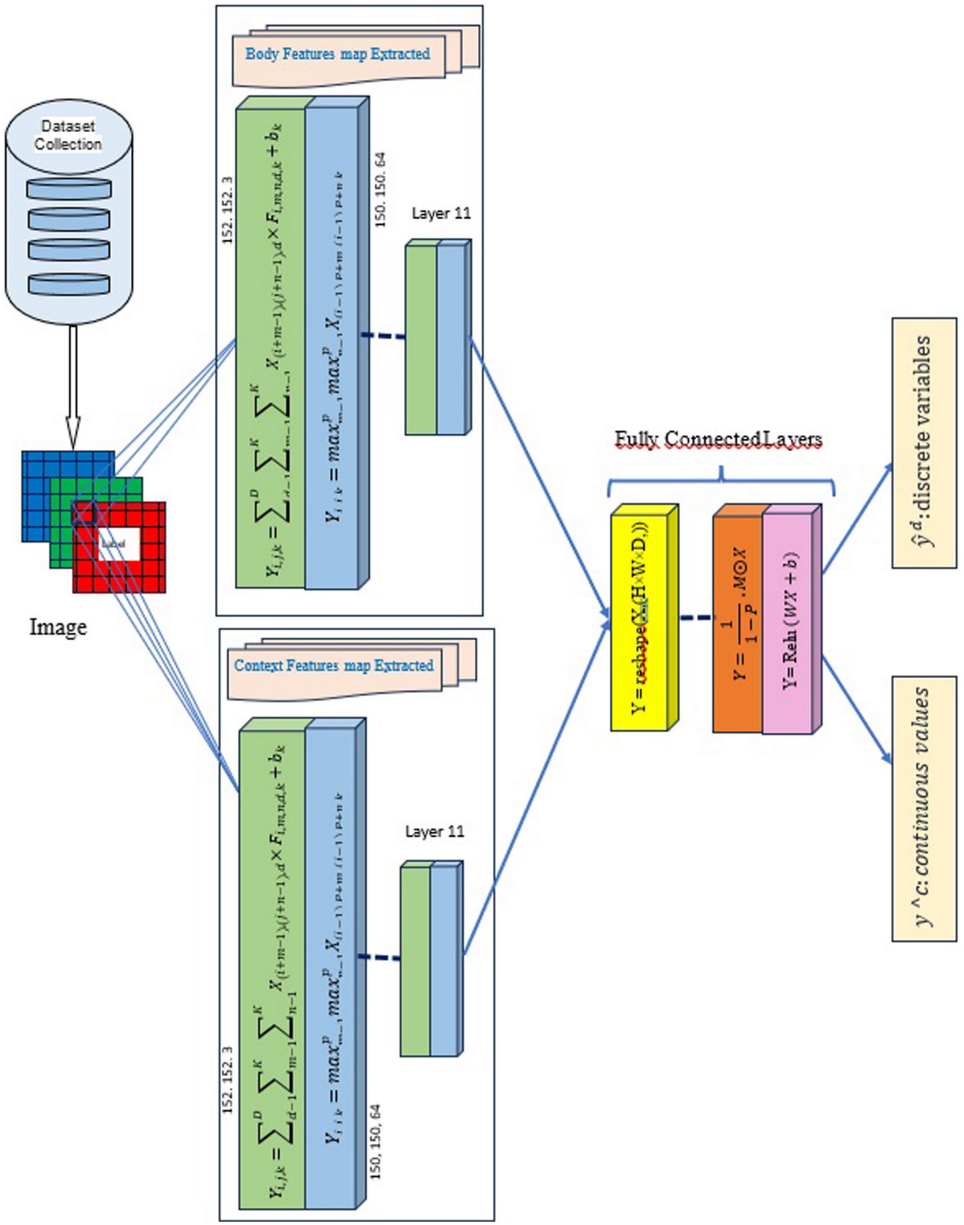
Architecture of the VGG Model: VGG19_context.

The VGG19_context model has been modified to enhance its adaptability to continuous dimensions. Specifically, our model presented in Figure 5 builds on the VGG19 architecture, adding custom dense layers for continuous dimensions. This model is designed to handle 150 × 150 pixel images and does not use pre-trained weights. It sequentially adds dense layers with ReLU activation, resulting in a softmax output layer that classifies images into 3 categories: Valence, Arousal, and Dominance. The model is compiled using the “adamax” optimizer and categorical cross-entropy loss, with a focus on training optimization through callbacks such as ModelCheckpoint and EarlyStopping. The use of dropout layers and dense layers in the model, albeit with different configurations and optimization approaches, underscores the versatility and adaptability of DCNN architectures in addressing various image classification tasks in emotion recognition.

### 4.4 Architecture of the concatenation model

**Figure 6:**
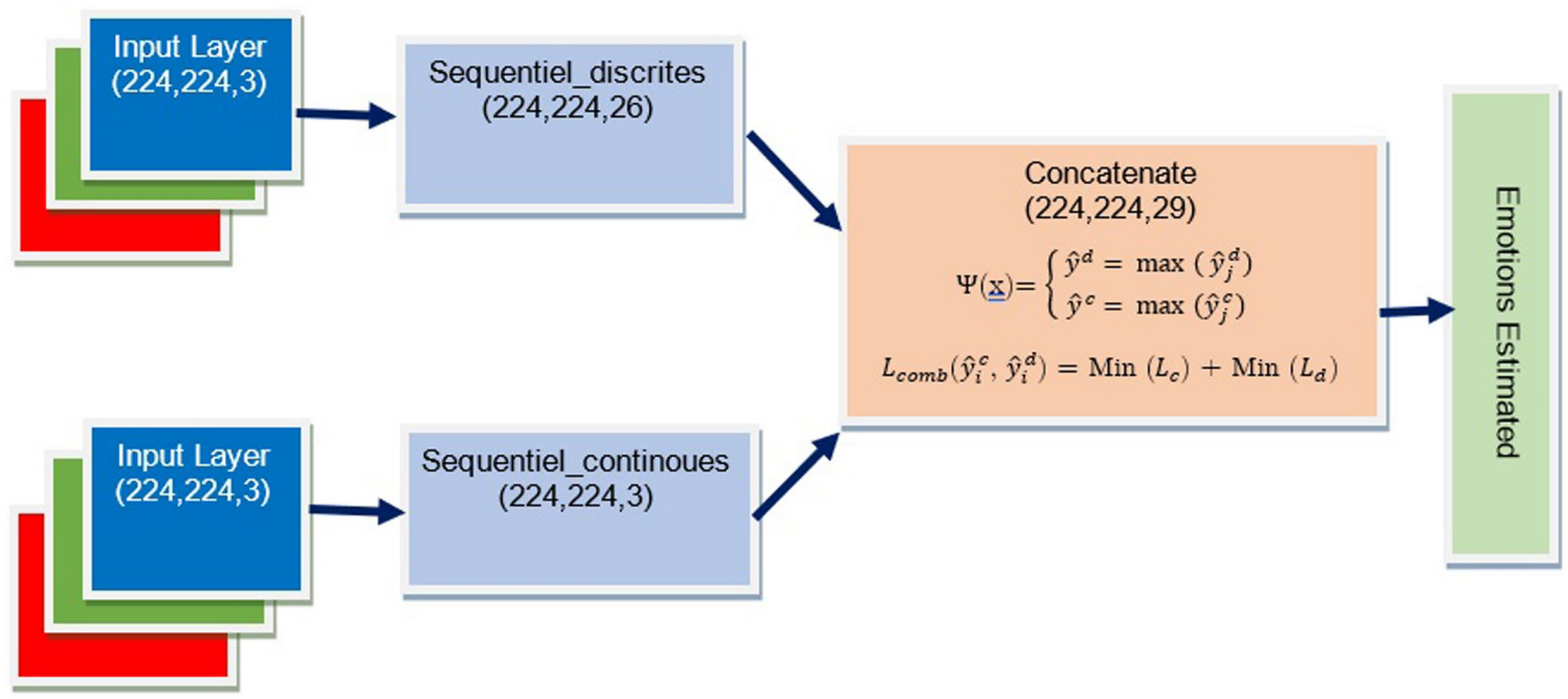
The architecture of the concatenation model.

Our concatenation model shown in Figure 6 is made up of two prediction models, namely the “Sentiment_recognition_model” for discrete variables and the “Sentiment_recognition_model” for continuous dimensions. The model requires two inputs, namely “input1” and “input2,” which are the predictions of discrete variables and continuous values made by the “Sentiment_recognition_model.” We have added a new dense layer consisting of 64 neurons using a ReLU activation function. To prevent overfitting, we have included a dropout layer with a rate of 0.5. Finally, the fused output goes through another dense layer that uses a softmax activation function to predict emotion classes and their corresponding probabilities.

Using Keras, we merge the two pre-trained models, VGG19_context for discrete variables and VGG19_context for continuous dimensions. This process involves loading both models, integrating their layers with new inputs, and merging their outputs. The resulting model is optimized for accuracy and improved performance on complex tasks.


$$softmax_{i}:x\to \frac{e^{x_{i}}}{\sum_{j=1}^{M}e^{x_{j}}}$$

$\forall x\in {\mathbb{R}}^{N}$ , and M = 29


$$\psi \left(x\right)=\left\{\begin{array}{c} \;{\hat{y}}^{d}=\max\;\left(\;{\hat{y}}_{j}^{d}\right)\nabla j\in \left\{1,\ldots M\right\}\\ {\hat{y}}^{c}=\max\;\left({\hat{y}}_{j}^{c}\right)\nabla j\in \left\{1,\ldots M\right\} \end{array}\right.$$

The new model is then created using the Keras function “Model” with inputs “input1” and “input2” and the output as the merged layer.

### 4.5 Experiments and results

**Figure 7:**
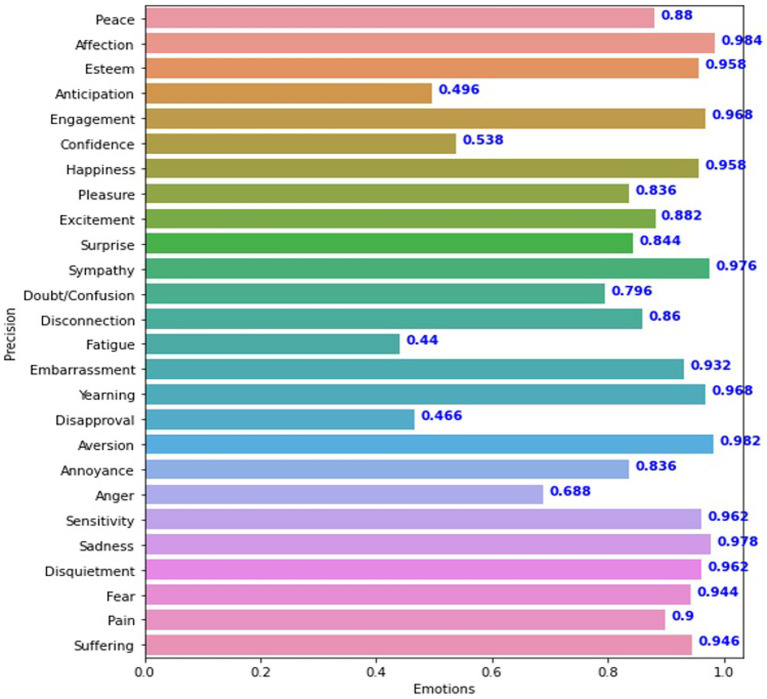
Precision of the Sentiment_recognition_model for each class of discrete variables.

This study used a deep convolutional neural network (DCNN) to detect emotions in discrete and continuous data. Model performance was evaluated using a variety of measures, including accuracy, mean accuracy, average accuracy, and mean accuracy (mAP). The VGG19_context model achieved an accuracy of 43.20% for continuous variables such as Valence, Arousal, and Dominance. The model’s overall performance was assessed using a categorical cross-entropy loss 21. The DCNN Sentiment_recognition_model performed similarly, with an accuracy of 43% and a categorical cross-entropy loss of 20, indicating that its accuracy and loss are comparable to those of the VGG19_context model. For the discrete variables in the EMOTIC dataset, the Sentiment_recognition model was optimized and refined using hyperparameter optimization, resulting in an average accuracy of 84.82% and an mAP of 78.39%. The Concatenation_model_DCNN combines body features and context features extracted by the Sentiment_recognition_model. It has an average precision of 68.1%. The Concatenation_model_VGG19 also combines body features and context features extracted by VGG19_context, with an average precision of 69.4%. Figure 7 shows the results of the DCNN model for each instance of the test set.

**Figure 8:**
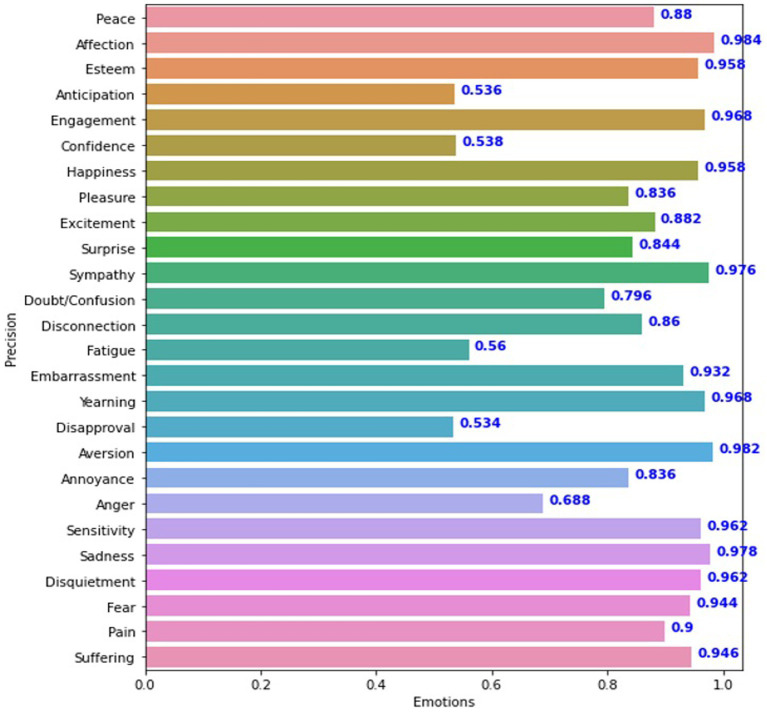
Presentation of Vgg19 model Precision for each feeling.

In comparison, the VGG19_context model, which used a more conventional approach, yielded a slightly better average accuracy of 85.42% and an mAP of 79.60, as seen in Figure 8. The results of these models suggest that while the VGG19_context model had a slight edge in performance for discrete variables, the Sentiment_recognition_model model’s performance was comparable, especially in continuous data scenarios. This comparison highlights the effectiveness of the Sentiment_recognition_model in handling diverse types of data in emotion recognition tasks. Figures 7,8 illustrating the results of both models provide a visual representation of their performance on the test sets.

The experiment produced an average accuracy of 43% for continuous dimensions. In contrast, Figure 7 summarizes the average accuracy of the Sentiment_recognition_model for discrete categories. The VGG19_context model showed promising results for discrete variables, as shown in FIGURE 8. These results are similar to those of the Sentiment_recognition model, for example, from the “Doubt/confusion,” “Fatigue,” “Disapproval,” and “Aversion” categories. Accuracy scores for the categories “Affection,” “Esteem,” “Commitment,” “Happiness,” “Sympathy,” “Embarrassment,” “Desire,” “Aversion,” “Sensitivity,” “Sadness,” “Worry,” “Fear” “Pain,” and “Suffering” ranged from 90 to 98% for both models, demonstrating the robust performance of the models in these areas.

## 5 Discussion

This section assesses the performance of models using different sets of features: body features alone, context features alone, and a combined model with both, as shown in Table 1. Our analysis indicates that both the DCNN and VGG19 models demonstrate similar levels of effectiveness when utilized with continuous variables, suggesting that the body and context feature sets provide a reliable foundation for interpretation in these scenarios. Notably, the similarity in performance metrics indicates that body and context features can independently capture the essential data to analyze continuous variables accurately.

**Table 1:** Performance comparison of sentiment recognition models.

**Model**	**mAP for continuous variables**	**mAP for discrete variables**	**Average accuracy**
DCNN (Sentiment_recognition_model)	43%	78.39%	84.82%
VGG19_context model	43.20%	79.60	85.42%
Concatenation_model_DCNN	68.1%		
Concatenation_model_VGG19	69.4%		

The body features used in the VGG19 model perform slightly better than the body features in the DCNN model for discrete variables. This improvement highlights contextual information’s importance in distinguishing discrete elements, suggesting that context adds valuable insights that body features alone may miss. The concatenation models (DCNN and VGG19) combine body and context features, leading to a significant increase in average accuracy compared to models using individual features. This notable improvement suggests that integrating diverse features captures a broader range of data nuances, paving the way for better overall performance and inspiring optimism about the potential of our research. The results reinforce the superiority of integrating both body and context features in sentiment recognition systems. While individual features provide a decent foundation, their combination offers a more accurate and robust approach. This holistic strategy is particularly effective in scenarios where high accuracy and detailed understanding are necessary, providing a solid basis for confidence in the effectiveness of concatenated models for enhanced performance.

We conducted a performance comparison of our approach with other state-of-the-art models on the EMOTIC datasets. Kosti et al. (2019) presented the basic EMOTIC model, consisting of two contexts: the background and body posture. Zhang et al. (2017) presented an efficient graph for contextual features that used regions proposed by the region proposal network (RPN) as nodes to construct a graph for the graph convolutional network (GCN). Our approach differs from these as we learn context by applying deep learning. We use two streams, one for extracting context features from the global image and the other for focusing on the principal agent’s body features. We then use a fusion network to merge the two streams and additional layers to predict discrete and continuous emotions. Our method extracts body features and context-specific information from the two streams of each modality. Table 2 shows the results of our technique and compares them with previous approaches using standard measures of precision and accuracy, with global classification accuracy as the evaluation metric:

**Table 2:** Performance comparison with other published methods on the EMOTIC dataset.

Emotion categories	Lee et al. (n.d.)	Kosti et al. (2019)	Li et al. (2018)	Zeng et al. (2020)	Hoang et al. (2021)	Our DCNN	Our VGG19
Peace	16.72	21.56	26.76	34.27	28.91	88.0	88.0
Affection	19.9.	2,785	37.93	45.23	44.48	98.4	98.4
Esteem	19.26	17.73	20.50	23.62	17.99	95.8	95.8
Anticipation	53.05	58.64	61.08	72.12	59.89	49.6	53.6
Engagement	46.58	87.53	88.12	91.12	88.71	96.8	96.8
Confidence	32.34	78.35	80.08	68.65	79.24	53.8	53.8
Happiness	49.36	58.26	76.01	74.71	83.02	95.8	95.8
Pleasure	19.47	45.46	55.64	65.53	55.47	83.6	83.6
Excitement	35.26	77.16	80.11	83.26	74.21	88.2	88.2
Surprise	10.92	18.81	17.92	17.37	16.27	84.4	84.4
Sympathy	17.12	14.71	15.26	34.28	15.37	97.6	97.6
Doubt/Confusion	28.98	29.63	33.50	35.12	25.42	79.6	79.6
Disconnection	22.80	21.32	28.32	43.12	34.24	86.0	86.0
Fatigue	13.04	09.70	17.51	16.23	22.62	44.0	56.0
Embarrassment	15.68	03.18	04.16	14.37	04.26	93.2	93.2
Yearning	09.79	08.34	10.11	14.29	14.04	96.8	96.8
Disapproval	16.04	14.97	21.54	19.82	24.54	46.6	53.4
Aversion	16.20	7.48	9.61	17.81	12.43	98.2	98.2
Annoyance	16.40	14.06	20.87	21.92	26.47	83.6	83.6
Anger	11.50	09.49	13.73	15.46	30.71	68.8	68.8
Sensitivity	10.34	09.28	09.59	08.32	15.89	96.2	96.2
Sadness	11.45	19.66	30.80	23.41	42.87	97.8	97.8
Disquietment	17.19	16.89	22.57	18.73	24.23	96.2	96.2
Fear	10.41	14.14	15.56	23.56	13.92	94.4	94.4
Pain	10.36	08.94	14.56	13.21	16.68	90.0	90.0
Suffering	11.68	18.84	30.70	26.39	46.23	94.6	94.6
mAP	20.84	27.38	32.41	35.48	35.16	78.39	79.6

Table 2 compares the performance of various models on the EMOTIC dataset. The comparison focuses on different emotion categories. The Sentiment_recognition_model DCNN and the VGG19_context model have proven to be significantly better than the models created by Kosti et al. (2019), Lee et al. (n.d.), and others in almost all categories when tested on the EMOTIC dataset. These models have shown exceptional accuracy in classifying emotions, consistently achieving accuracy rates above 80% and beyond 90%. Although models developed by Zeng et al. (2020) and Hoang et al. (2021) have shown better results, they still need to catch up to the high standards set by the Sentiment_recognition_model and VGG19_context models. Models like those created by Lee et al. and Kosti et al. often need help to achieve accuracy rates below 30% in several categories.

Disparities in the accuracy of emotion detection models, especially for emotions like “Affection,” “Esteem,” “Happiness,” “Sympathy,” and “Sadness,” can be attributed to variations in training procedures, data management techniques, or model architectures. Using more complex regularization techniques, reliable training datasets, and advanced feature extraction techniques would significantly improve the accuracy of these models. To demonstrate their efficacy, the Sentiment_recognition_model and VGG19_context model have total mean Average Precision (mAP) scores of 78.39 and 79.6, respectively, which are significantly higher than the next best score of 35.48 by Zeng et al. However, the DCNN and VGG19 models perform well in continuous emotion classes like Valence, Arousal, and Dominance. The VGG19_context model shows a slightly higher mean average precision (mAP) in these categories. Our method is effective in examining various features of the scene context in the image estimated prediction, compared with Ding and Tao (2018).

Table 3 compares actual and estimated predictions for discrete categories and continuous emotions for a few images. These predictions do not match the actual predictions in the Table 3 provided. For example, for row 2, the actual prediction for the cropped image of the principal agent includes the discrete category “Engagement,” and the associated continuous emotions are Valence: 7, Arousal: 4, and Dominance: 7 Whereas the estimated prediction is “Fatigue” with probability:1, it also includes the discrete category “Doubt/Confusion” with very low probability (1. 2004818342e-32) and “Disapproval” with a low probability: 1.951509602e-35, which does not correspond to the actual prediction “Commitment.” The continuous emotion is “arousal” with a probability of 1. Furthermore, given that our model provides the maximum probability class of discrete values and the maximum probability continuous dimension, the result is fatigue and arousal.

**Table 3:** Comparison of actual and estimated predictions regarding discrete categories and continuous emotions for some images.

Images	Cropped image of the main agent	Real prediction		Estimated prediction probability	
		Discrete categories	Continuous emotions	Discrete categories	Continuous emotions
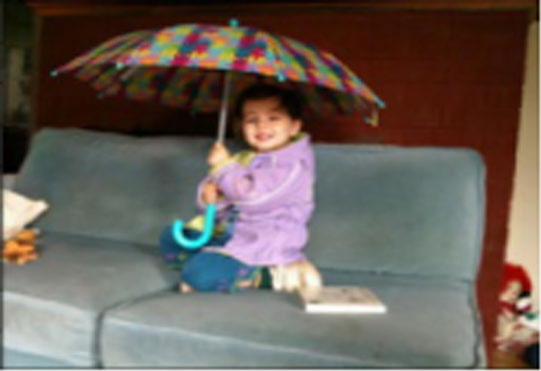	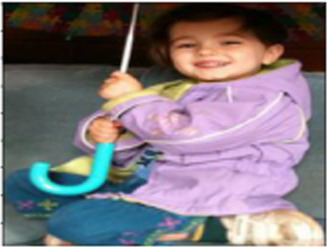	Engagement	Valence: 7.0 Arousal: 4.0 Dominance: 7.0	Doubt/Confusion: 1.2004818342e-32 Fatigue: 1.0 Disapproval: 1.951509602e-35	Arousal: 1.0
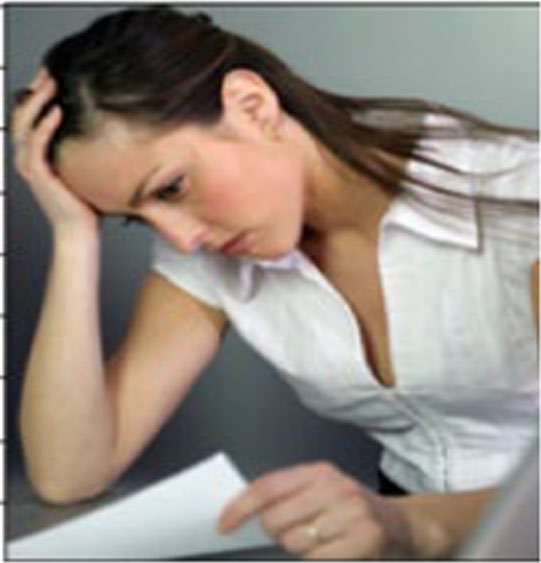	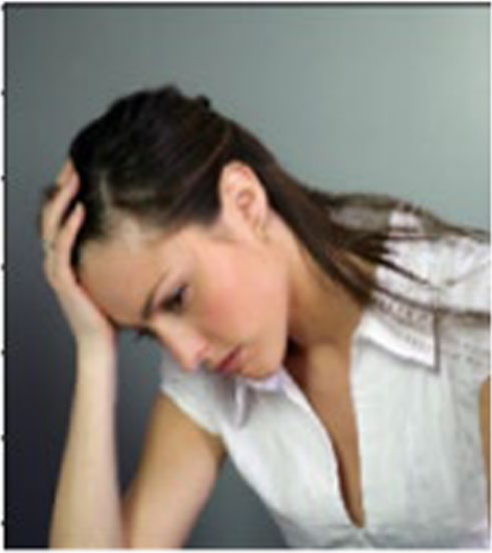	Excitement Happiness Peace Pleasure Anticipation Confidence Engagement Surprise	Valence: 5.5 Arousal: 6.5 Dominance: 7.0	Fatigue: 1.0	Dominance: 1.0
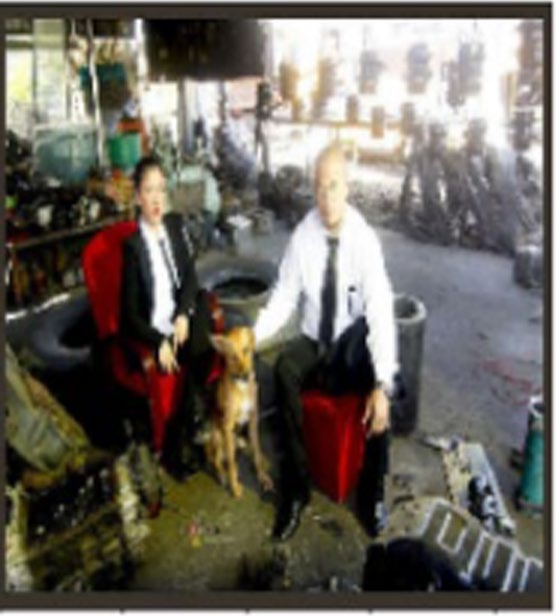	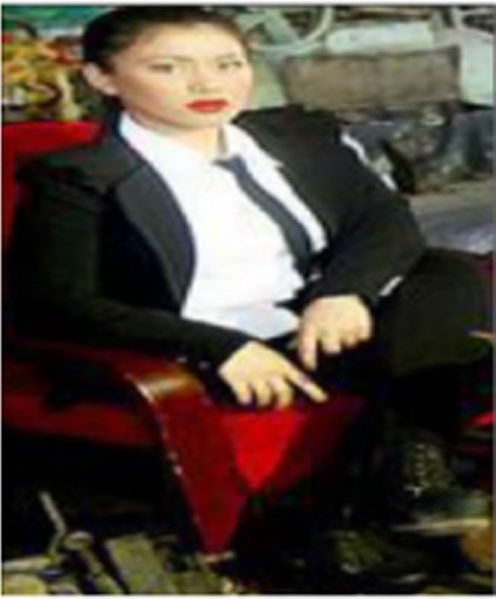	Affection Disconnection Engagement Esteem Peace Sympathy Anger Aversion Pleasure	Valence: 4.5 Arousal: 2.0 Dominance: 7.5	Fatigue: 1.0 Disapproval: 1.4502387481e-35	Dominance: 1.0
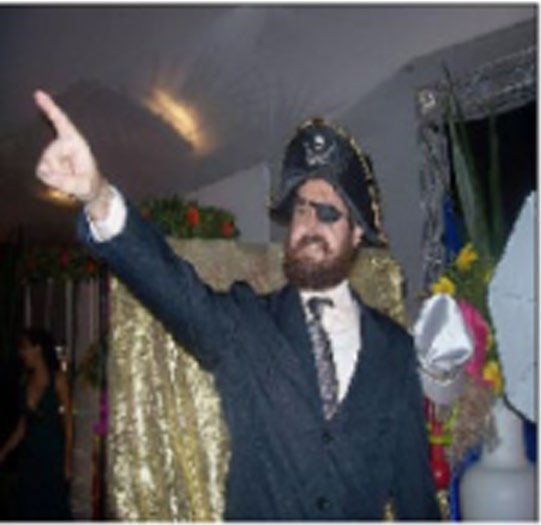	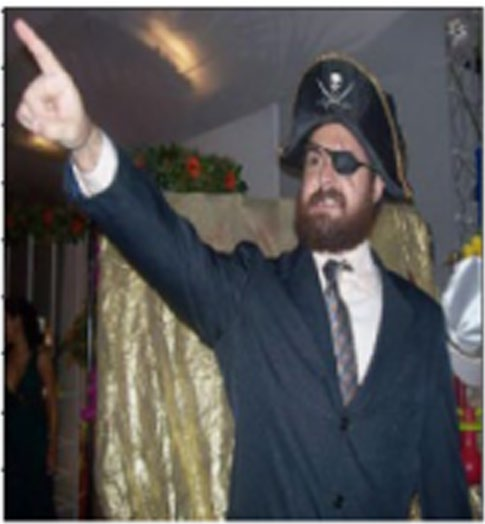	Excitement Happiness Peace Pleasure Anticipation Confidence Engagement Surprise	Valence: 5.5 Arousal: 6.5 Dominance: 7.0	Doubt/Confusion: 4.832917599e-31 Fatigue: 1.0 Disapproval: 7.67900693e-30	Valence: 2.827515602e-37 Dominance: 1.0
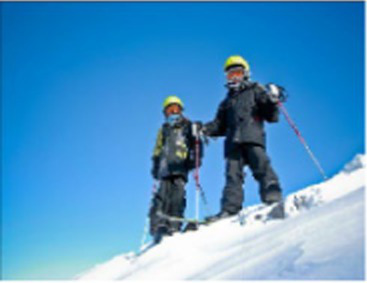	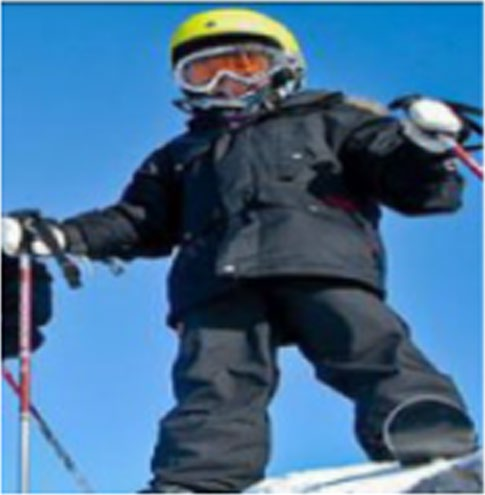	Anticipation Peace	Valence: 7.0 Arousal: 7.0 Dominance: 8.0	Fatigue: 1.0	Dominance: 1.0
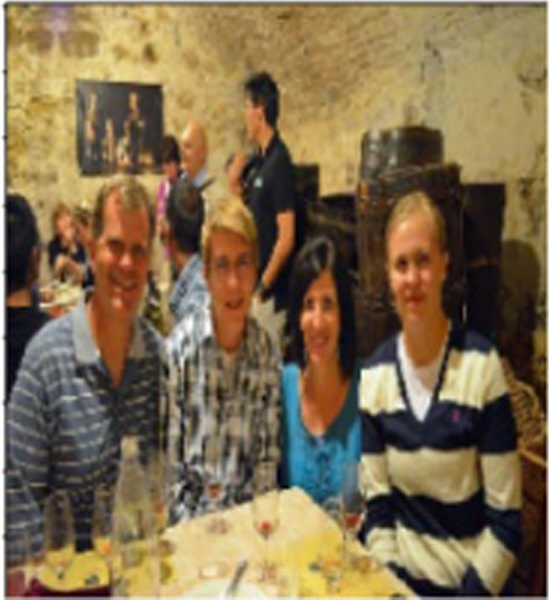	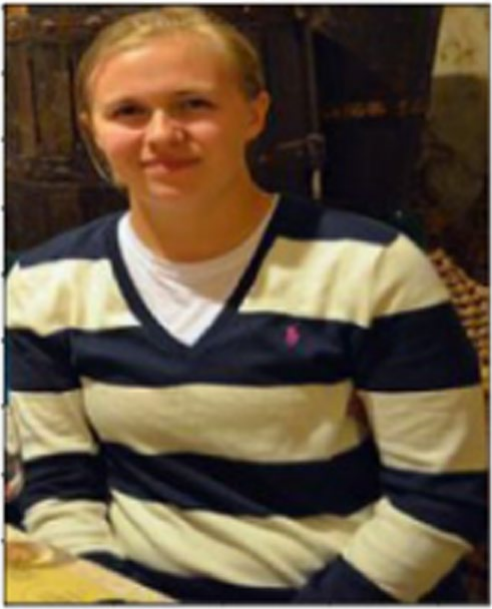	Excitement Happiness Peace Pleasure Anticipation Confidence Engagement Surprise	Valence: 5.5 Arousal: 6.5 Dominance: 7.0	Sympathy: 1.0 Fatigue: 1.0 Disapproval: 5.744446490e-38	Valence: 5.304753457e-35 Dominance: 1.0

Images 1, and 3-6 are taken from Papers With Code- Frames Dataset (hyperlink: https://paperswithcode.com/dataset/frames-dataset), licensed under CC BY-SA. Image 2 is taken from the Emotic Dataset (hyperlink: https://paperswithcode.com/dataset/emotic), licensed under CC BY-SA.

The variable performance of different emotional categories in the discrete variable analysis raises several important considerations. Firstly, the unique characteristics of each emotional category should be examined to understand why certain emotions are more challenging to detect. Secondly, the impact of the contexts or scenarios in which the images were captured should be studied, as factors such as lighting and social environment can significantly affect the visibility of expressions and, therefore, recognition accuracy. Thirdly, potential biases in the training data that might favour certain emotions should be evaluated, as an unbalanced data distribution can distort model performance. Fourthly, it is important to recognize that some emotions are inherently more complex or ambiguous, making them more prone to misclassification. Lastly, interindividual variability in emotional expression should be considered, as it can lead to inconsistent recognition between different people.

This analysis highlights the model’s ability to predict a range of emotions with specific probabilities. It demonstrates its performance in accurately identifying both discrete and continuous emotional states. Table 3 is crucial for evaluating the model’s effectiveness in emotion recognition. It provides insight into its predictive capabilities and accuracy in different emotional contexts.

## 6 Conclusion

This study addresses the issue of contextual recognition of emotional states. We use images taken in natural environments of real people in real situations from the EMOTIC dataset (MSCOCO, Ade20k) and images from the Emodb_small and Framesdb datasets. We create two new models, the first DCNN type and the second based on the VGG19 architecture, to identify emotions in context (body and context) by merging the 26 emotional categories identified and described in this study with the three frequent, continuous emotional dimensions for estimating emotions in context. These models serve as a basis for answering the question of predicting emotional states in context. They are built on deep learning techniques and optimizing our models’ hyperparameters to improve performance. The proposed pipeline is then completed by merging our models.

We conducted an examination of a method for recognizing emotions based on context. However, the models sometimes fail to accurately predict how an object will affect a person’s emotions due to image brightness, position, size, and clarity. Our research achieved an mAP of 78.39% for the Sentiment_recognition_model DCNN model and an mAP of 79.60% for VGG19_context. These results show potential for advancing contextual emotion recognition in images beyond previous findings. Our positive results have significant implications for emotional computing, human-robot communication, social robotics, and human-computer interaction, opening up new opportunities for technological development and better collaboration between humans and robots. Future research should focus on creating large and diverse datasets that include various emotional expressions from different demographics and cultures, which would help develop more robust and generalizable models. However, we must explore more sophisticated models that can incorporate contextual information, such as attention mechanisms or graph neural networks, to improve the accuracy of emotion detection in complex scenarios.

Our research has revealed the potential benefits of affective computing and social robots, leading to more intuitive and emotionally sophisticated human-robot interaction. This advancement may transform the way humans and robots interact meaningfully and cooperatively. We are also interested in the untapped potential of gaze tracking for context-based emotion assessment, especially when combining contextual emotions with the facial emotions of the principal agent.

## Data availability statement

The datasets presented in this study can be found in online repositories. The names of the repository/repositories and accession number(s) can be found at: https://github.com/fatiha5/emotions.

## Author contributions

FL: Conceptualization, Data curation, Formal analysis, Funding acquisition, Investigation, Methodology, Project administration, Resources, Software, Supervision, Validation, Visualization, Writing – original draft, Writing – review & editing. BH: Conceptualization, Data curation, Formal analysis, Funding acquisition, Investigation, Methodology, Project administration, Resources, Software, Supervision, Validation, Visualization, Writing – original draft, Writing – review & editing. RO: Conceptualization, Data curation, Formal analysis, Funding acquisition, Investigation, Methodology, Project administration, Resources, Software, Supervision, Validation, Visualization, Writing – original draft, Writing – review & editing.
