# Corrigendum: Contextual emotion detection in images using deep learning

**DOI:** 10.3389/frai.2024.1476791

**Published:** 2024-09-03

**Authors:** Fatiha Limami, Boutaina Hdioud, Rachid Oulad Haj Thami

**Affiliations:** Advanced Digital Enterprise Modeling and Information Retrieval (ADMIR) Laboratory, Rabat IT Center, Information Retrieval and Data Analytics Team (IRDA), ENSIAS, Mohammed V University in Rabat, Rabat, Morocco

**Keywords:** computer vision, contextual recognition, EMOTIC, emotion recognition, body language, human-robot communication

In the published article, there was an error in affiliation. Instead of “Advanced Digital Enterprise Modeling and Information Retrieval (ADMIR) Research Laboratory, Information Retrieval and Data Analytics Team (IRDA), ENSIAS, Mohammed V University, Rabat, Morocco”, the affiliation should read “Advanced Digital Enterprise Modeling and Information Retrieval (ADMIR) Laboratory, Rabat IT Center, Information Retrieval and Data Analytics Team (IRDA), ENSIAS, Mohammed V University in Rabat, Rabat, Morocco”.

The authors apologize for this error and state that this does not change the scientific conclusions of the article in any way. The original article has been updated.

